# Breast cancer incidence and case fatality among 4.7 million women in relation to social and ethnic background: a population-based cohort study

**DOI:** 10.1186/bcr3086

**Published:** 2012-01-06

**Authors:** Omid Beiki, Per Hall, Anders Ekbom, Tahereh Moradi

**Affiliations:** 1 Division of Epidemiology, Institute of Environmental Medicine, Karolinska Institutet, Box 210, SE-171 77, Stockholm, Sweden; 2 Kermanshah University of Medical Sciences, Kermanshah, Iran; 3 Department of Epidemiology and Biostatistics, Karolinska Institutet, PO Box 281, SE-171 77, Stockholm, Sweden; 4 Unit of Clinical Epidemiology, Department of Medicine, SOLNA, Karolinska Institutet, SE-171 76, Stockholm, Sweden

## Abstract

**Introduction:**

Incidence of breast cancer is increasing around the world and it is still the leading cause of cancer mortality in low- and middle-income countries. We utilized Swedish nationwide registers to study breast cancer incidence and case fatality to disentangle the effect of socioeconomic position (SEP) and immigration from the trends in native Swedes.

**Methods:**

A nation-wide cohort of women in Sweden was followed between 1961 and 2007 and incidence rate ratio (IRR) and hazard ratio (HR) with 95% confidence intervals (CIs) were estimated using Poisson and Cox proportional regression models, respectively.

**Results:**

Incidence continued to increase; however, it remained lower among immigrants (IRR = 0.88, 95% CI = 0.86 to 0.90) but not among immigrants' daughters (IRR = 0.97, 95% CI = 0.94 to 1.01) compared to native Swedes. Case fatality decreased over the last decades and was similar in native Swedes and immigrants. However, case fatality was significantly 14% higher if cancer was diagnosed after age 50 and 20% higher if cancer was diagnosed in the most recent years among immigrants compared with native Swedes. Women with the highest SEP had significantly 20% to 30% higher incidence but had 30% to 40% lower case fatality compared with women with the lowest SEP irrespective of country of birth. Age at immigration and duration of residence significantly modified the incidence and case fatality.

**Conclusions:**

Disparities found in case fatality among immigrants by age, duration of residence, age at immigration and country of birth emphasize the importance of targeting interventions on women that are not likely to attend screenings or are not likely to adhere to the therapy suggested by physicians. The lower risk of breast cancer among immigrant women calls for more knowledge about how the lifestyle factors in these women differ from those with high risk, so that preventative measures may be implemented.

## Introduction

Breast cancer is the most common tumor among women worldwide. However, there is large geographical variation in its incidence; with the exception for Japan, the incidence ranks highest in high-income countries [1]. More than half of the incident cases in the world occur in Europe and North America [2]. The incidence of breast cancer has been increasing since the 1970s even in countries with a reported low rate, such as Japan, Korea, India and even Africa which lacks accurate population data [2]. A Westernized life-style, including older age at giving birth to a first child and fewer children, are among the explanations for the increasing incidence seen worldwide [3].

Despite the substantial improvement in breast cancer prognosis and survival, it is still the leading cause of cancer mortality in low- and middle-income countries and more than half of the breast cancer mortality is reported from low- and middle-income countries [4].

Migrant studies are classical tools for exploring the importance of environmental, social and genetic factors in the etiology of diseases and has been particularly important for disentangling the etiology of cancer [5]. Migrant studies have also been performed to explore differences in mortality, if any, among immigrants and the host country. Migration from low- to high-incidence countries, particularly if migration takes place at young ages [6,7], has been shown to influence both incidence and mortality from breast cancer. Differences found in these studies might be explained by differences in biologic and pathologic characteristics of cancer, quality of medical care, such as delays in follow-up after abnormal screening, and disparities in the receipt of cancer treatment.

In this large, nationwide cohort study, we utilized Sweden's established system of demographic and medical population-based registers to explore the impact of country of birth and social position on breast cancer incidence and case fatality among large and growing immigrant populations and their daughters in Sweden and among native Swedes.

## Materials and methods

### Database

The cohort was built through linkages between Swedish national registers using personal identity numbers (PIN). PIN is a 10-digit number which is maintained by the National Tax Board office for all individuals who have resided longer than one year in Sweden since 1947 [8]. The linkages have been completed by Statistics Sweden and the Centre for Epidemiology at the National Board of Health and Welfare.

For the purpose of this study, we used: 1) The Swedish Cancer Registry, which was founded in 1958 and covers the whole population of Sweden. It is compulsory for every health care provider to report newly detected cancer cases diagnosed at clinical, pathological or other laboratory examinations, as well as cases diagnosed at autopsy to the registry. The overall completeness of the registry is high and close to 100% [9]; 2) The National Population and Housing Censuses cover demographic, occupational and socioeconomic factors, such as income, occupation and education for the total population of Sweden between 1960 and 1990. This practice ended in 1990 [10] and was substituted by Longitudinal Integration Database for Health Insurance and Labor Market studies (LISA by Swedish acronym). LISA is a yearly-updated nationwide database consisting of data from 1990 and onwards on all individuals 16 years or older registered as living in Sweden [11]. We obtained individual information on highest level of education from these two registers; 3) the Multi-Generation Register, where we obtained information on reproductive history as well as the links between parents and children. The register consists of all individuals born in 1932 or later who were registered in Sweden sometime after 1961 [12]; 4) The Cause of Death Register, where the information on cause-specific mortality was obtained. The number of non-reported cases in this register is low and previous studies support the use of this register as an appropriate source of breast cancer death in Sweden [13]; and 5) The Swedish Population Register, including the country of birth of the Swedish population [14]. To ensure confidentiality, the PIN was replaced by serial numbers through Statistics Sweden. We have obtained permission to use the databases and registries we used in our study from the Regional Board of The Ethical Committee, Stockholm (Dnr: 2005/726-31 and amendment 2009/587-32).

### Classification of country of birth, socio-economic position, and covariates

We classified foreign-born individuals into six groups by the continents. We further subdivided continents into world regions, as defined by the United Nations Population Division. We report pooled data for countries and regions when we did not have enough power. For detailed information about the final classification, please refer to Tables 1, 2, 3, 4, 5,6. We classified study participants into three groups: i) women born outside of Sweden, called immigrants, ii) women born in Sweden with at least one parent born outside of Sweden, called immigrants' daughters, and iii) Sweden-born women with both parents born in Sweden, called native Swedes. For persons who had no registration of the parental country of birth, it was assumed that the parents originated from the same country as their child.

**Table 1:** Incidence rate ratio of breast cancer by country of birth, 1961 to 2007

Birth region	**No**.	PYRS^$^	IRR* (95% CI)	Birth region	**No**.	PYRS^$^	IRR* (95% CI)
**All immigrants**	8,853	12,056	**0.88 (0.86 to 0.90)**	Poland	474	582	**0.79 (0.73 to 0.87)**
**Africa**	131	427	**0.64 (0.54 to 0.76)**	Romania	98	112	0.89 (0.73 to 1.09)
***Eastern/Middle***	65	269	**0.55 (0.43 to 0.70)**	Soviet Union†	151	168	0.94 (0.80 to 1.10)
Eritrea	14	38	0.71 (0.42 to 1.19)	***Northern***	4,496	5,059	**0.93 (0.90 to 0.96)**
Ethiopia	26	91	0.71 (0.48 to 1.05)	Denmark	460	468	0.98 (0.90 to 1.08)
Other	25	140	**0.40 (0.27 to 0.59)**	Estonia	136	114	0.93 (0.78 to 1.10)
***Northern***	46	82	0.87 (0.65 to 1.16)	Finland	3,321	3,753	**0.93 (0.90 to 0.97)**
Egypt	12	15	0.97 (0.55 to 1.70)	Iceland	29	46	1.18 (0.82 to 1.70)
Morocco	20	34	0.88 (0.57 to 1.37)	Latvia	27	23	1.09 (0.75 to 1.59)
Other	14	32	0.78 (0.46 to 1.32)	Norway	427	528	**0.84 (0.76 to 0.92)**
***Southern***	6	9	0.89 (0.40 to 1.98)	UK	87	108	0.94 (0.76 to 1.16)
South Africa	6	8	1.02 (0.46 to 2.26)	Other	9	18	0.72 (0.37 to 1.38)
Other	0	1	NA	***Southern***	1,077	1522	**0.85 (0.80 to 0.90)**
***Western***	14	67	**0.49 (0.29 to 0.82)**	Bosnia	250	343	**0.78 (0.69 to 0.89)**
				Greece	102	161	0.84 (0.69 to 1.01)
**Asia**	961	2,392	**0.73 (0.69 to 0.79)**	Italy	50	57	1.03 (0.78 to 1.35)
***Eastern***	69	281	**0.63 (0.50 to 0.80)**	Portugal	30	33	1.18 (0.82 to 1.68)
China	31	85	**0.58 (0.41 to 0.83)**	Spain	40	52	0.91 (0.67 to 1.24)
Japan	23	30	0.80 (0.53 to 1.21)	Yugoslavia†	604	872	**0.84 (0.78 to 0.91)**
Korea Rep.	11	152	**0.53 (0.29 to 0.96)**	Other	1	4	0.32 (0.05 to 2.29)
Other	4	14	0.55 (0.20 to 1.46)	***Western***	801	751	0.93 (0.87 to 1.00)
***South-Central***	310	722	**0.78 (0.69 to 0.87)**	Austria	84	76	1.07 (0.86 to 1.33)
India	27	146	0.74 (0.50 to 1.07)	France	33	41	0.94 (0.67 to 1.32)
Iran	252	412	**0.86 (0.76 to 0.98)**	Germany	612	562	**0.91 (0.84 to 0.98)**
Sri Lanka	14	70	0.63 (0.37 to 1.07)	Netherlands	39	42	0.97 (0.71 to 1.32)
Other	17	94	**0.34 (0.21 to 0.55)**	Switzerland	25	20	1.29 (0.87 to 1.91)
***South-Eastern***	109	376	**0.53 (0.44 to 0.64)**	Other	8	10	0.89 (0.45 to 1.78)
Philippines	34	72	0.74 (0.53 to 1.04)				
Thailand	38	159	**0.47 (0.34 to 0.65)**	***Latin America***	245	588	**0.65 (0.57 to 0.74)**
Viet Nam	23	106	**0.40 (0.27 to 0.61)**	Argentina	25	28	1.15 (0.78 to 1.70)
Other	14	38	0.64 (0.38 to 1.08)	Bolivia	10	23	0.72 (0.39 to 1.33)
***Western***	473	1014	**0.78 (0.71 to 0.85)**	Brazil	18	38	0.78 (0.49 to 1.24)
Iraq	198	324	1.01 (0.87 to 1.17)	Chile	105	286	**0.52 (0.43 to 0.63)**
Lebanon	64	166	0.84 (0.66 to 1.08)	Peru	21	38	0.78 (0.51 to 1.20)
Syria	82	130	0.95 (0.76 to 1.18)	Uruguay	25	28	1.12 (0.75 to 1.65)
Turkey	106	334	**0.48 (0.40 to 0.58)**	Other	41	147	**0.61 (0.45 to 0.83)**
Other	23	60	**0.64 (0.43 to 0.97)**				
				***North America***	84	112	0.90 (0.73 to 1.12)
***Europe***	7,426	8,518	**0.91 (0.89 to 0.93)**	Canada	9	16	0.71 (0.37 to 1.36)
***Eastern***	1,052	1,186	**0.87 (0.82 to 0.92)**	USA	75	96	0.94 (0.75 to 1.17)
Bulgaria	26	33	0.91 (0.62 to 1.34)				
Czechoslovakia	92	108	**0.79 (0.65 to 0.97)**	***Oceania***	6	19	0.61 (0.27 to 1.36)
Hungary	211	181	1.07 (0.93 to 1.22)				

* IRRs are adjusted for age at follow-up and calendar period of follow-up, education and place of residence at diagnosis. Native Swedish women with both parents born in Sweden were the reference group. **$ **PYRS = person-years at risk divided by 1000. **† **Czechoslovakia includes Czech Republic and Slovakia. Soviet Union includes Belarus, Moldova, Russian Federation and Ukraine. Yugoslavia includes Croatia, Macedonia, Serbia, Slovenia and Montenegro.

Highest attained level of education was used as a surrogate indicator for socio-economic position and categorized into four levels (< 9, 10 to 12, 13+ years, and unknown).

We stratified our analysis by age at exit (< 50 and 50+ years), calendar period of follow-up with respect to incidence rate (1961 to 1985, 1986 to 1995, 1996 to 2000, and 2001 to 2007) and calendar period of diagnosis with respect to case fatality (1961 to 1985, 1986 to 1995, 1996 to 2000, and 2001 to 2007) and geographical region of diagnosis (Gothenburg, Linkoping, Lund-Malmo, Stockholm, Umeå, and Uppsala) where each of the six Swedish national Oncologic Centrum is placed. In an attempt to study the possible influence of lifestyle and environmental exposures, we stratified the immigrants by age at immigration (younger than 15 years, 15 to 34, and 35 years or older) and duration of residence in Sweden (less than 5 years, 5 to 14, 15 to 29, and 30 years or longer).

### Incidence cohort and statistical analysis

There were 4,749,611 women registered in the Swedish Population Register who were born after 1 January 1930 and lived in Sweden at any time during 1 January 1961 and 31 December 2007. We excluded women with an unknown birthplace (0.03%), with a history of breast cancer before the start date of the study (0.01%), for whom we found a death date (0.01%) or an emigration date (2.5%) before entry into the cohort (January 1961, date of birth or first immigration date or their first appearance in census, whichever occurred last).

The final cohort was followed from 1 January 1961, date of birth or first immigration date for immigrants, whichever occurred last, until they exited from the cohort, which was the date of diagnosis of breast cancer (ICD-7 code: 170 Malignant Neoplasm of Breast), first emigration date, death or end of follow-up (31 December 2007), whichever came first.

We calculated the incidence rate ratios (IRRs) with 95% confidence intervals (CIs) using Poisson regression models. All analyses were adjusted for age at follow-up (0 to 14, 15 to 29, 30 to 34, 35 to 39, 40 to 44, 45 to 49, 50 to 54, 55 to 59, 60 to 64, 65 to 69 and 70+) and calendar period of follow-up (1961 to 1965,......, 2001 to 2005, 2006 to 2007.

### Case fatality cohort and statistical analysis

The outcomes of interests were death due to any cause and death due to breast cancer as the underlying cause of death. In all, 76,152 women were diagnosed with primary invasive breast cancer. To avoid inclusion of cases detected by autopsy but not registered as such, we excluded 259 (0.34%) women who died within one month of diagnosis.

Breast cancer patients were followed from date of diagnosis until date of death, first emigration date, or end of follow-up (31 December 2007), whichever came first. In breast cancer-specific case fatality analysis, the patient's follow-up was censored if either death for other reasons or emigration took place. Hazard ratios (HRs) with 95% confidence intervals (CIs) for breast cancer patients were calculated using stratified Cox proportional hazards regression model. Point estimates and 95% CIs were produced using the maximum partial likelihood for the effect estimates. The validity of the proportional hazards assumption was evaluated using a martingale residual based graphical and numerical approach.

## Results

### Incidence

Our cohort comprised 4,553,484 women, of which 760,214 (16.7%) were immigrants, 495,917 (10.9%) were immigrants' daughters and 3,297,353 (72.4%) were native Swedish women. We observed 76,152 cases of breast cancer during 133 million person-years of follow-up in our cohort. Immigrants (52.4, SD ± 10.2) and native Swedish women (53.2, SD ± 10.0) had a similar age at diagnosis. Age at diagnosis among immigrants' daughters was on average 48.6 (SD ± 9.4). Mean age at immigration was 22.6 (SD ± 13.4) years ranking from the highest among immigrants from Bosnia (29.1, SD ± 16.4) and lowest among immigrants from the Republic of Korea (6.3, SD ± 11.2). Mean duration of residence was 15.9 (SD ± 4.8) years, ranking from the highest among immigrants from Austria (28.3, SD ± 21.1) and the lowest among immigrants from China (6.9, SD ± 7.1).

Overall, immigrants had lower incidence (IRR = 0.88, 95% CI = 0.86 to 0.90) of breast cancer while their daughters had a similar incidence (IRR = 0.97, 95% CI = 0.94 to 1.01) compared with native Swedes (Tables 1 and 2).

**Table 2:** Incidence rate ratio (IRR) of breast cancer among immigrants' daughters in Sweden, 1961 to 2007

	Cases	PYRS^$^	IRR* (95% CI)	
			Vs. native Swedes	Vs. mothers
**All immigrants' daughters**	2,808	11,457	0.97 (0.94 to 1.01)	**1.08 (1.03 to 1.12)**
**Africa**	7	340	0.60 (0.29 to 1.25)	0.60 (0.27 to 1.30)
***Northern ***	4	136	1.00 (0.37 to 2.65)	1.46 (0.50 to 4.28)
***Africa except Northern***	3	206	0.39 (0.13 to 1.21)	0.39 (0.12 to 1.27)
**Asia**	31	1,039	1.10 (0.78 to 1.57)	1.18 (0.82 to 1.71)
***Eastern ***	15	82	1.60 (0.96 to 2.65)	**2.09 (1.17 to 3.71)**
***South-Central ***	7	245	0.84 (0.40 to 1.76)	0.88 (0.41 to 1.90)
***South-Eastern ***	3	133	1.11 (0.36 to 3.44)	1.71 (0.53 to 5.55)
***Western ***	6	592	0.78 (0.35 to 1.74)	0.53 (0.23 to 1.23)
**Europe**	2,538	9,590	0.97 (0.93 to 1.01)	1.04 (0.99 to 1.09)
***Eastern ***	227	901	0.97 (0.85 to 1.10)	1.09 (0.94 to 1.27)
Czechoslovakia^†^	28	121	0.91 (0.63 to 1.31)	1.15 (0.70 to 1.87)
Hungary	36	219	0.88 (0.63 to 1.22)	1.00 (0.65 to 1.53)
Poland	66	349	0.88 (0.69 to 1.12)	1.09 (0.84 to 1.42)
Soviet Union^†^	96	171	1.15 (0.94 to 1.40)	1.14 (0.87 to 1.49)
Other	10	60	1.22 (0.66 to 2.28)	1.20 (0.62 to 2.33)
***Northern ***	1,902	6,628	0.96 (0.92 to 1.01)	1.01 (0.95 to 1.08)
Denmark	332	963	0.99 (0.89 to 1.11)	1.01 (0.86 to 1.19)
Estonia	144	329	0.97 (0.82 to 1.14)	1.34 (0.96 to 1.89)
Finland	830	3,920	0.94 (0.88 to 1.01)	0.98 (0.90 to 1.07)
Latvia	22	53	0.91 (0.60 to 1.39)	0.96 (0.47 to 1.93)
Norway	556	1,230	0.96 (0.88 to 1.04)	1.14 (0.99 to 1.31)
UK	36	184	1.08 (0.78 to 1.50)	1.12 (0.75 to 1.68)
Other	8	43	1.13 (0.57 to 2.27)	1.01 (0.46 to 2.21)
***Southern ***	54	989	0.85 (0.65 to 1.11)	0.87 (0.65 to 1.16)
Italy	23	137	0.99 (0.66 to 1.48)	0.95 (0.51 to 1.78)
Yugoslavia^†^	15	545	0.62 (0.37 to 1.02)	0.69 (0.40 to 1.19)
Other	16	331	0.95 (0.58 to 1.56)	0.86 (0.50 to 1.45)
***Western ***	418	1,338	1.01 (0.92 to 1.11)	1.12 (0.97 to 1.30)
Austria	48	166	1.01 (0.76 to 1.34)	0.82 (0.53 to 1.24)
France	21	75	1.13 (0.74 to 1.74)	1.37 (0.75 to 2.52)
Germany	326	970	1.04 (0.93 to 1.16)	**1.26 (1.07 to 1.50)**
Netherlands	20	84	0.95 (0.62 to 1.48)	0.80 (0.43 to 1.47)
Other	12	60	0.70 (0.40 to 1.23)	**0.48 (0.23 to 0.98)**
**Latin America**	12	272	1.22 (0.69 to 2.15)	**2.00 (1.11 to 3.60)**
**North America**	228	369	1.04 (0.91 to 1.19)	1.03 (0.79 to 1.35)
Canada	11	32	0.92 (0.51 to 1.67)	1.23 (0.47 to 3.19)
USA	217	336	1.05 (0.92 to 1.20)	0.99 (0.75 to 1.31)
**Oceania**	4	16	**3.26 (1.22 to 8.69)**	**5.34 (1.16 to 24.60)**

* All IRRs are adjusted for age at follow-up, calendar period of follow-up, years of education and place of residence at diagnosis. Native Swedish women with both parents born in Sweden were the reference group. **^$^**PYRS = person-years at risk divided by 1,000. **^† ^**Czechoslovakia includes Czech Republic and Slovakia. Soviet Union includes Belarus, Moldova, Russian Federation and Ukraine. Yugoslavia includes Croatia, Macedonia, Serbia, Slovenia and Montenegro.

Except for Northern America and Oceania, immigrants born in all other continents were at significantly lower incidence of breast cancer compared to native Swedes, with women born in Africa having the lowest incidence (IRR = 0.64; 95% CI 0.54 to 0.76) followed by immigrants from Latin America (IRR = 0.65; 95% CI 0.57 to 0.74) (Table 1). Within the African continent, women born in eastern, middle and western regions had statistically, significantly half the incidence of native women. Immigrants from all regions in Asia had statistically significantly 20 to 50% lower incidence compared with native Swedes. Among immigrant women born in this part of world, women born in Thailand, Vietnam and Turkey had the lowest statistically significant incidence compared with native Swedes. Within Europe, there was significantly lower incidence (10 to 15%) for women born in eastern, northern and southern regions. The incidence was significantly lower for women born in the former Czechoslovakia (IRR = 0.79; 95% CI 0.65 to 0.97), Poland (IRR = 0.79; 95% CI 0.73 to 0.87), Finland (IRR = 0.93; 95% CI 0.90 to 0.97), Norway (IRR = 0.84; 95% CI 0.76 to 0.92), Bosnia (IRR = 0.78; 95% CI 0.69 to 0.89), Germany (IRR = 0.91; 95% CI 0.84 to 0.98), and former Yugoslavia (IRR = 0.84; 95% CI 0.78 to 0.91). Within Latin America, women born in Chile had statistically, significantly 50% lower incidence compared with native Swedes. Women born in all other studied countries had similar incidence of breast cancer compared with Native Swedes (Table 1).

As shown in Table 2 a convergence toward the incidence of native Swedes was observed among immigrants' daughters. Immigrants' daughters from all continents had similar incidence of breast cancer compared with native Swedes, except for those with either one or both parents born in Oceania that showed a significantly higher incidence (IRR = 3.26, 95% CI = 1.22 to 8.69). Immigrants' daughters, with either one or both parents born in Eastern Asia (IRR = 2.09, 95% CI = 1.17 to 3.71), Latin America (IRR = 2.00, 95% CI = 1.11 to 3.60), and Oceania (IRR = 5.34, 95% CI = 1.16 to 24.60), had a significantly higher incidence of breast cancer compared with their mothers. At the country level, immigrants' daughters with parents born in Germany (IRR = 1.26, 95% CI = 1.07 to 1.50) had significantly higher incidence of breast cancer compared with their mothers. However, lack of statistical power hindered any definitive conclusion because of wide confidence intervals at the country level.

Irrespective of background, women with the highest educational level had significantly higher incidence of breast cancer compared to those with lower education (Table 3). The incidence was 20% to 30% higher among women with the highest versus lowest educational level.

**Table 3:** Incidence rate ratio (IRR) in Sweden by education, calendar year and area of diagnosis

	Immigrants			Immigrants' daughters			Native Swedes		
	Cases	PYRS^$^	IRR* **(95% CI)**	Cases	PYRS^$^	IRR* **(95% CI)**	Cases	PYRS^$^	IRR* **(95% CI)**
**Years of education**									
13+	2,142	2,661	**1.28 (1.21 to 1.36)**	1,029	3,010	**1.18 (1.06 to 1.32)**	17,963	3,0772	**1.18 (1.16 to 1.21)**
10 to 12	3,365	4,572	**1.14 (1.08 to 1.20)**	1,292	4,521	1.07 (0.96 to 1.19)	26,595	4,6316	**1.05 (1.03 to 1.07)**
0 to 9	2,866	3,529	Reference	478	1,641	Reference	19,644	2,3095	Reference
Unknown	480	1,296	1.02 (0.93 to 1.13)	9	2,286	0.99 (0.50 to 1.89)	289	8,864	**1.41 (1.26 to 1.58)**
**Calendar period of follow-up**									
1961 to 1985	1,069	3,738	**0.83 (0.75 to 0.91)**	145	3,992	0.82 (0.66 to 1.01)	8,321	48,239	**0.75 (0.73 to 0.78)**
1986 to 1995	2,123	2,991	**0.87 (0.81 to 0.92)**	565	2,826	0.92 (0.81 to 1.03)	16,687	25,836	**0.87 (0.85 to 0.89)**
1996 to 2000	1,848	2,003	**0.89 (0.84 to 0.95)**	674	1,769	1.05 (0.96 to 1.16)	14,046	14,169	**0.95 (0.93 to 0.97)**
2001 to 2007	3,813	3,328	Reference	1,424	2,871	Reference	25,437	20,803	Reference
**Area of residence at diagnosis**									
Gothenburg	1,641	2,391	**0.93 (0.87 to 0.98)**	543	2,313	0.89 (0.80 to 1.00)	11,548	21,632	**0.83 (0.81 to 0.85)**
Linkoping	687	950	1.00 (0.92 to 1.09)	201	884	0.89 (0.76 to 1.04)	7,341	12,762	**0.87 (0.84 to 0.89)**
Lund-Malmo	1,569	1,993	1.05 (0.98 to 1.11)	437	1,730	0.99 (0.88 to 1.11)	12,003	18,074	0.99 (0.97 to 1.02)
Stockholm	2,906	3,898	Reference	839	3,497	Reference	12,992	20,870	Reference
Umea	460	648	0.94 (0.85 to 1.03)	250	827	**0.79 (0.69 to 0.91)**	6,653	12,301	**0.80 (0.77 to 0.82)**
Uppsala	1,590	2,131	0.97 (0.91 to 1.03)	538	2,123	0.90 (0.81 to 1.00)	13,954	23,171	**0.89 (0.87 to 0.91)**

* All IRRs are adjusted for age at follow-up and calendar period of follow-up and the other two variables shown. **^$^**PYRS = person-years at risk divided by 1,000.

**Figure 1:**
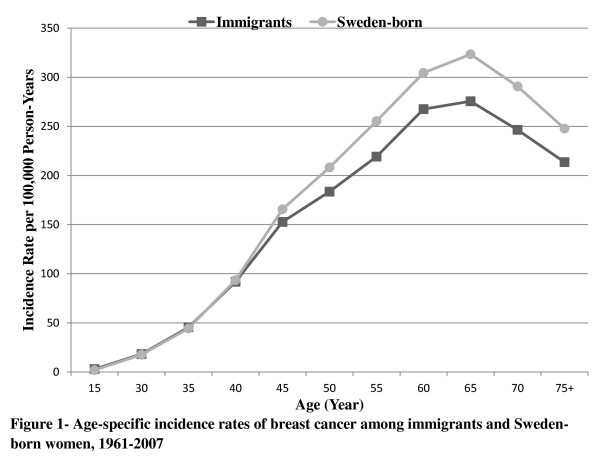


The incidence rate of breast cancer increased to a maximum at age 65 years and then dropped among both immigrants and Sweden-born women. The differences in rates between immigrants and Sweden-born women increased by increasing age at diagnosis (Figure 1).

**Figure 2:**
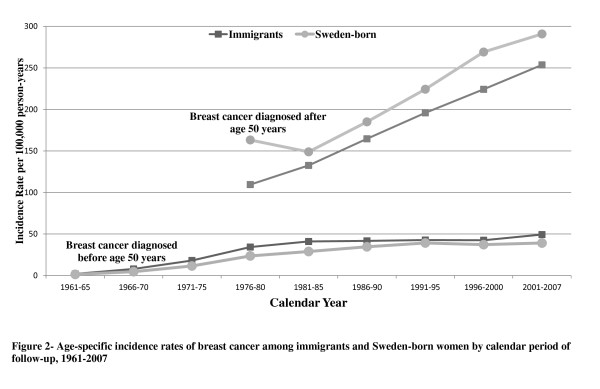


Age-specific incidence rates stratified by age at breast cancer diagnosis before and after age 50 are presented in Figure 2. While rates increased by increasing calendar period of follow-up, higher differences of incidence rates between immigrants and Sweden-born women were found in most recent years.

To find whether calendar period of follow-up has any effect on the incidence of breast cancer, we divided our results into four categories of calendar period follow-up. The incidence increased slightly with increasing year of follow-up in that incidence was 20% higher during most recent years; 2001 to 2007, compared with incidence during 1961 to 1985. When we stratified results by place of residence at diagnosis, we found those who were residing in Stockholm at diagnosis had a higher incidence of breast cancer compared with those from other areas in Sweden. However, results were statistically significant only among native Swedes (Table 3).

Age at immigration and duration of residence significantly altered the incidence of breast cancer (Table 4). When stratifying the results by age at immigration we found a statistically significant decrease in the incidence by increasing age at immigration among all immigrants as one group (Table 4). This increase was more pronounced among immigrants from low-risk countries in Africa, Asia and Eastern Europe (results not shown). When stratifying by duration of residence, however, we found an overall 10% lower incidence among immigrants who stayed less than 30 years compared with those who stayed longer in Sweden (Table 4).

**Table 4:** Incidence rate ratio (IRR) among immigrants by age at immigration and duration of residence, 1961-2007

	Incidence of breast cancer	
	
	Cancer	IRR* (95% CI)
**Age at immigration**		
0 to 14	835	**0.85 (0.77 to 0.93)**
15 to 34	5,276	1.02 (0.96 to 1.08)
35+	1,702	Reference
**Duration of residence**		
0 to 4	660	**0.88 (0.79 to 0.98)**
5 to 14	1,523	**0.91 (0.84 to 0.98)**
15 to 29	2,557	**0.92 (0.87 to 0.98)**
30+	3,073	Reference

* All IRRs are adjusted for age at follow-up, calendar period of follow-up and region of origin.

### Case fatality

The final cohort included 75,893 women with breast cancer, of which 8,818 (11.6%) were immigrants, 2,800 (3.7%) were immigrant's daughters and 64,275 (84.7%) were native Swedes.

We observed a total of 14,024 deaths due to breast cancer among 75,893 women with breast cancer; 1,578 death in immigrants, 446 in immigrants' daughters and 12,000 in native women. Women with more education had a better survival compared to women with less education (data not shown in table), irrespective of country of birth. Women with the lowest educational level had around 30% statistically, significantly higher risk of dying from breast cancer compared with women with the highest level of education (data not shown in table).

We found improving survival over calendar years among native Swedes and immigrants' daughters (Table 5). However, immigrants whose cancer was diagnosed in more recent years (2001 to 2007) had a significantly higher risk (HR = 1.20, 95% CI = 1.01 to 1.43) of dying from breast cancer compared with native Swedes, while those with cancer diagnosed in earlier years (1960 to 2000) had a similar risk compared with native Swedes (Table 5).

**Table 5:** Hazard ratio (HR) of case fatality of breast cancer by education, calendar year and area of diagnosis

	Immigrants		Immigrants' daughters	
	Death	HR* (95% CI)	Death	HR* (95% CI)
**Years of education**				
13+	285	1.01 (0.88 to 1.17)	144	0.98 (0.80 to 1.20)
10 to 12	542	0.99 (0.90 to 1.09)	202	0.98 (0.83 to 1.15)
0 to 9	621	1.03 (0.94 to 1.13)	94	0.85 (0.67 to 1.09)
Unknown	130	0.92 (0.59 to 1.44)	6	0.65 (0.17 to 2.51)
**Calendar period of diagnosis**				
1961 to 1985	444	0.98 (0.87 to 1.10)	49	0.84 (0.59 to 1.19)
1986 to 1995	615	0.94 (0.86 to 1.04)	192	0.96 (0.82 to 1.13)
1996 to 2000	282	1.10 (0.95 to 1.27)	129	0.90 (0.73 to 1.10)
2001 to 2007	237	**1.20 (1.01 to 1.43)**	76	1.10 (0.83 to 1.45)
**Age at diagnosis**				
0 to 50	132	0.92 (0.85 to 1.00)	82	0.94 (0.83 to 1.08)
50+	551	**1.14 (1.04 to 1.25)**	112	0.96 (0.78 to 1.18)
**Area of residence at diagnosis**				
Gothenburg	314	1.03 (0.90 to 1.18)	88	1.16 (0.91 to 1.48)
Linkoping	132	1.02 (0.83 to 1.26)	22	0.78 (0.47 to 1.31)
Lund-Malmo	274	1.09 (0.94 to 1.26)	86	0.86 (0.66 to 1.11)
Stockholm	476	0.97 (0.87 to 1.09)	124	0.88 (0.72 to 1.09)
Umea	103	0.96 (0.75 to 1.23)	44	1.07 (0.73 to 1.57)
Uppsala	279	0.98 (0.85 to 1.13)	82	0.95 (0.74 to 1.22)

* All HRs are adjusted for age at follow-up and calendar period of diagnosis. HRs are also mutually adjusted for years of education and place of residence at diagnosis if applicable. Native Swedish women defined as women with both parents born in Sweden.

We further observed an increasing risk of dying due to breast cancer by decreasing age at diagnosis, irrespective of birth country. The risk was 25% higher if breast cancer was diagnosed at an age younger than 50 (HR = 1.24, 95% CI = 1.18 to 1.29) than that if cancer was diagnosed at age 50 or older (data not shown in table). In addition, immigrants whose cancer was diagnosed after age 50 had a significantly higher risk (HR = 1.14, 95% CI = 1.04 to 1.25) of dying from breast cancer, while those with cancer diagnosed before age 50 had a lower risk (HR = 0.92, 95% CI = 0.85 to 1.00) compared with natives (Table 5).

Compared with Stockholm, all regions had similar case fatality except for Umeå, where the case fatality was higher (Table 5).

When we stratified breast cancer case fatality by country of birth, we found a similar case fatality for most immigrants compared with native Swedes (Table 6). The risk of dying due to breast cancer, however, was 2.5 times higher among immigrants born in Northern Africa (HR = 2.81, 95% CI = 1.13 to 6.96). Analysis of immigrants' daughters at the country level was hampered by lack of power and was, therefore, not included.

**Table 6:** Hazard ratio (HR) in Sweden by country of birth, 1961 to 2007

Birth region	Death	HR* (95% CI)	Birth region	Death	HR* (95% CI)
**All immigrants**	1578	1.01 (0.95 to 1.07)	Other	4	**3.25 (1.06 to 9.95)**
			***Northern***	840	1.02 (0.94 to 1.11)
**Africa**	27	1.10 (0.67 to 1.82)	Denmark	90	1.13 (0.90 to 1.43)
***Eastern/Middle***	17	0.81 (0.43 to 1.52)	Estonia	27	0.70 (0.45 to 1.11)
Ethiopia	8	0.97 (0.37 to 2.52)	Finland	612	1.04 (0.95 to 1.14)
Other	9	0.74 (0.32 to 1.70)	Iceland	3	2.49 (0.67 to 9.29)
***Northern***	7	**2.81 (1.13 to 6.96)**	Latvia	6	0.61 (0.24 to 1.55)
***Southern***	2	0.48 (0.05 to 4.23)	Norway	90	0.96 (0.76 to 1.21)
***Western***	1	N/A	UK	12	0.71 (0.35 to 1.44)
			***Southern***	192	0.95 (0.80 to 1.11)
**Asia**	120	0.91 (0.72 to 1.14)	Bosnia	36	1.40 (0.91 to 2.13)
***Eastern***	7	0.73 (0.31 to 1.74)	Greece	24	0.90 (0.57 to 1.41)
***South-Central***	40	0.93 (0.63 to 1.35)	Italy	5	0.95 (0.38 to 2.34)
India	5	0.54 (0.19 to 1.52)	Portugal	8	0.50 (0.21 to 1.16)
Iran	31	0.97 (0.63 to 1.51)	Spain	5	0.59 (0.21 to 1.67)
Other	4	1.57 (0.54 to 4.53)	Yugoslavia^†^	114	0.95 (0.77 to 1.17)
***South-Eastern***	14	0.98 (0.49 to 1.98)	***Western***	175	1.02 (0.86 to 1.21)
Thailand	5	0.30 (0.07 to 1.29)	Austria	16	1.43 (0.82 to 2.47)
Other	9	2.02 (0.90 to 4.53)	France	7	1.00 (0.39 to 2.57)
***Western***	59	0.91 (0.65 to 1.28)	Germany	139	1.00 (0.83 to 1.21)
Iraq	19	0.76 (0.43 to 1.34)	Netherlands	9	1.21 (0.56 to 2.62)
Lebanon	4	0.76 (0.23 to 2.52)	Other	4	0.59 (0.21 to 1.69)
Syria	13	0.93 (0.38 to 2.24)			
Turkey	16	1.05 (0.56 to 1.97)	**Latin America**	29	1.06 (0.67 to 1.66)
Other	7	1.64 (0.57 to 4.77)	Chile	14	1.45 (0.81 to 2.61)
			Uruguay	4	0.90 (0.19 to 4.38)
**Europe**	1,388	1.01 (0.95 to 1.08)	Other	11	0.72 (0.33 to 1.56)
***Eastern***	181	1.05 (0.88 to 1.24)			
Czechoslovakia^†^	18	1.07 (0.62 to 1.85)	**North America**	13	1.03 (0.49 to 2.16)
Hungary	36	0.92 (0.63 to 1.34)	Canada	2	0.71 (0.15 to 3.30)
Poland	89	1.26 (0.98 to 1.61)	USA	11	1.18 (0.50 to 2.77)
Romania	16	0.71 (0.41 to 1.24)			
Soviet Union^†^	18	0.81 (0.47 to 1.39)	**Oceania**	1	0.45 (0.06 to 3.36)

* HRs are adjusted for age at follow-up and calendar period of diagnosis, education and place of residence at diagnosis. Native Swedish women with both parents born in Sweden were the reference group. **^† ^**Czechoslovakia includes Czech Republic and Slovakia. Soviet Union includes Belarus, Moldova, Russian Federation and Ukraine. Yugoslavia includes Croatia, Macedonia, Serbia, Slovenia and Montenegro.

When stratifying the results by age at immigration we found an overall similar HR among immigrants who immigrated at ages younger than 35 compared with those who immigrated at older ages (Table 7). However, we found statistically significant risk modification by age at immigration among women from low-risk countries in Africa, Asia and Eastern Europe (data not shown in table). We also found an overall statistically significant higher case fatality among immigrants who stayed less than 30 years compared with those who stayed longer in Sweden (Table 7).

**Table 7:** Hazard ratio (HR) of breast cancer case fatality by age at immigration and duration of residence.

	Breast cancer-case fatality	
	
	Death	HR* (95% CI)
**Age at immigration**		
0 to 14	88	0.73 (0.501.05)
15 to 34	716	0.92 (0.71 to 1.19)
35+	240	Reference
**Duration of residence**		
0 to 4	134	**1.14 (0.72 to 1.80)**
5 to 14	272	**1.52 (1.08 to 2.13)**
15 to 29	363	**1.34 (1.06 to 1.71)**
30+	275	Reference

* All HRs are adjusted for age at follow-up, calendar period of diagnosis and region of origin.

## Discussion

In this large, nation-wide cohort study among women with diagnosis of invasive neoplasm of the breast in Sweden, we found that women with the most education, as an indicator of socio-economic position, had statistically, significantly 20% to 30% higher incidence of breast cancer, but 30% to 40% better breast cancer survival compared with women with the lowest educational level irrespective of country of birth. Furthermore, our study showed increasing breast cancer incidence over the last decades in native Swedes and immigrants, albeit not in immigrants' daughters. We found immigrant women overall had a lower incidence of breast cancer than native Swedes with the lowest risk, almost half that of native Swedes, observed among women born in China, South Korea, Thailand, Viet Nam, Turkey and Chile

There are a number of known risk factors for breast cancer; high socioeconomic status [15,16], radiation exposure [17], diethylstilbestrol exposure during pregnancy [18], low age at menarche and high age at menopause [19], postmenopausal high body mass index [20], and long term use of hormone replacement therapy [21] have been associated with increased risk of breast cancer while low age at first childbirth [19], high parity [19] and physical activity [15,22,23] have been associated with lower risk of breast cancer. In general, about 90% of the breast cancer cases in high income countries are attributed to hormone level-related factors [24]. The low incidence found in this study among immigrant women, apart from the borderline significant decreased risk among some groups, such as immigrants from Finland and Germany, could partially be attributed to differences in distribution of breast cancer risk factors in comparison to native women. We lacked information on individual risk factors, the clinical stage and histological grade. We should point out that the observed small significant absolute differences between immigrants and Sweden-born women, for example, among immigrants from Finland, might be due to the large number of populations under study.

Our finding of younger age at diagnosis among immigrants' daughters could simply be due to the younger age of the population at risk in this group.

Our finding of the convergence of incidence towards the Swedish incidence level was observed among immigrants' daughters, particularly among those whose parents were from low-risk areas, such as Asia and Latin America. This is in agreement with studies on immigrants from Asia and Latin America in the US and immigrants from Ireland in the UK [6,25,26]. A significant variation in incidence by race and ethnicity and strong scientific support has been accumulating for the fact that immigrants undergo changes in breast cancer risk after migration, mostly due to modifiable environmental and behavioral factors [6,27]. The level of acculturation, measured by language use or duration of residence, has been shown to be inversely associated with age at menarche, number of pregnancies and duration of breastfeeding; and has been positively linked to age at first full-term pregnancy, obesity, screening attendance and health care utilization [28-30]. In our study, we examined changes in risk with respect to three indicators of acculturation, that is, age at immigration, duration of residence and generation in Sweden. Previous studies on cancer among immigrants in Sweden neither focused on breast cancer *per se *nor considered age at immigration and duration of residence [31-34]. Some studies highlighted the importance of exposures, such as diet and residential history. Among adult immigrants from low-risk areas, place of birth acted as a protective factor, while breast cancer incidence was shown to increase among the younger migrants [27]. Our findings of risk modification by age at immigration among women from low-risk countries in Africa, Asia and Eastern Europe and by duration of residence, in line with studies on Italian migrants and US Hispanics, suggest that the timing of migration might be a strong predictor of breast cancer incidence, and highlights the importance of life style factors [28,35,36].

In this study, we found that women with the most education had statistically significantly 30% to 40% better breast cancer survival compared with women with the lowest educational level, irrespective of country of birth. Furthermore, our study showed decreasing breast cancer case fatality over the last decades in native Swedes and immigrants but not in immigrants' daughters. We found disparities in breast cancer case fatality by age at diagnosis and calendar period of diagnosis. Immigrants whose cancer was diagnosed after age 50 or in 2001 to 2007 had higher breast cancer case fatality compared with corresponding native Swedes.

Our findings of increasing incidence [37-39] and improving survival [40] over time were similar to the results of previous studies conducted in Sweden. These studies, however, were confined to women living in Sweden without considering their immigrant status. Our findings of disparity in breast cancer case fatality between immigrants and native Swedes are in line with the results of studies in the US [41,42]. The decrease in breast cancer case fatality is probably reflecting the better prognosis and, thus, increasing survival of breast cancer cases [43]. Factors such as advances in therapy and earlier detection through the implementation of screening programs are suggested elements responsible for the better prognosis [44].

The disparities we found between immigrants and native Swedes by age and calendar period of diagnosis are novel. These disparities might be due to lack of absorbance in the screening program among older and recently arrived immigrants. Establishment of mammography screening in Sweden has progressed from a pilot study in 1974 through clinical trials to service screening [45,46]. Screening with mammography for early detection of breast cancer has been provided by all Sweden's 26 county councils since 1997. It took 23 years from the initial pilot study through clinical trials to the establishment of mammography service screening throughout Sweden. Mammography outside screening programs, clinical mammography, is available throughout Sweden. A negative relation between the use of clinical mammography and participation in the screening programs has been noticed [47]. Previous studies in Sweden have found several socio-economic and health behavior-related factors that predict non-attendance in mammographic service screening programs and found that non-attendees are at higher risk for advanced breast cancer [48-50]. Two other studies have shown that immigrants, especially those from non-Nordic countries, were more than twice as likely to be non-attendees compared with Swedish-born women [51,52]. These results are in agreement with our findings and warrant further attention for adhering migrant population to mammographic screening. We found overall similar case fatality in immigrants, immigrants' daughters and native Swedes. Case fatality increased significantly by increasing age at immigration and decreased significantly by increasing duration of residence. It was also statistically, significantly higher among immigrants if cancer was diagnosed after age 50 or in most recent years.

Inequalities in case fatality within different education groups among both immigrants and Swedes found in this study is in line with the cumulative evidence from different epidemiological studies, including studies from Sweden with equal access to a uniform health care system, indicating higher breast cancer mortality among socio-economically disadvantaged women [53-56].

It has already been shown that breast cancer mortality is lower in Sweden than in Denmark [57]. In contrast, we found a similar mortality among immigrants from Denmark and native Swedes, which supports the conclusion by Jensen and colleagues emphasizing the importance of early breast cancer detection in Sweden on decreasing breast cancer mortality [58]. Our findings of higher breast cancer survival by increasing age is in line with the results of previous Swedish studies implying that young women affected by breast cancer have higher mortality even if diagnosed early and receiving intensive treatment [59,60]. In addition, we found higher mortality among immigrant than native women if they were diagnosed at older ages. The older immigrant women in Sweden, thus, suffer from higher mortality for two reasons: first, their old age; since later diagnosis in older women has been associated with worse mortality in Sweden [59,60], and second, their migration status. The differences in management and screening uptake have already been observed among immigrant women compared to natives and might be an explanation for this finding [61-63].

Different pathological stage at diagnosis and biological markers, including estrogen, progesterone and HER2 receptor status, have been indicated as probable factors responsible for disparities found among different ethnic and socio-economic groups in US and Sweden [64-68]. There are studies that have shown significant differences in the frequency of estrogen/progesterone positivity between Vietnamese and Swedish breast cancer patients [69] and between different major racial/ethnic groups in the US [70-72]. We found that some part of these discrepancies in case fatality might be due to different distribution of these factors among the study population. We had no access to individual data on these factors and were not able to consider them in our analysis. A limitation of our study, which applies to almost all migrant studies, is the possibility of selection bias. The population of interest is likely to be non-random and thus may not represent the populations of origin. In addition, forces of selection probably differ from population to population. International immigration is feasible more among women with higher education and socio-economic position, factors which are known to be associated with the risk of breast cancer. Immigrants from different countries listed in this study vary by reasons for immigration and selection forces. Part of the variations we found among the immigrants could be due to these differences. These limitations need special consideration when comparisons are made among countries of birth and call for studies with focus on each specific group to disentangle the effect of selection forces from environmental factors on breast cancer risk and case fatality.

The major strength of our study is the population-based design with a long follow-up of all native Swedes and foreign-born women during the study period. Because information on exposure was collected before the diagnosis of cancer, misclassification with regards to exposure is unlikely and, if any, is most likely independent of breast cancer and thus, non-differential.

## Conclusions

In conclusion, our finding of convergence of breast cancer incidence towards the Swedish level among immigrants' daughters plus effect-modification by age at immigration and duration of residence among immigrants indicates the importance of pre-migration factors, and highlights the importance of early-life exposures. Disparities found in cases of fatality among immigrants when stratifying results by age and duration of residence, age at immigration and country of birth emphasize the importance of designing and implementing active interventions in order to reduce incidence and, particularly, fatality in susceptible sub-groups of the female population. In addition, the lower risk of breast cancer among immigrant women outlines the importance of understanding how lifestyle factors in these women differ from those with high risk, so that preventative measures may be implemented.

## Abbreviations

CI: confidence interval; HR: hazard ratio; ICD-7: International Classification of Disease, Seventh Edition; IRR: incidence rate ratio; LISA: Database for Health Insurance and Labor Market studies; PIN: personal identity number; SEP: socioeconomic position.

## Competing interests

The authors declare that they have no competing interests.

## Authors' contributions

TM and OB had full access to all of the data in the study and take responsibility for the integrity of the data and the accuracy of the data analysis. OB and TM conceived and designed the study. TM acquired the data. Analysis and interpretation of data were undertaken by OB, PH, AE and TM. OB drafted the manuscript. Critical revision of the manuscript for important intellectual content was conducted by OB, PH, AE and TM. Statistical analysis was conducted by OB. TM obtained funding. All authors read and approved the final manuscript.
